# The Association between Education and Work Stress: Does the Policy Context Matter?

**DOI:** 10.1371/journal.pone.0121573

**Published:** 2015-03-26

**Authors:** Thorsten Lunau, Johannes Siegrist, Nico Dragano, Morten Wahrendorf

**Affiliations:** 1 Centre for Health and Society, Institute for Medical Sociology, Medical Faculty, University of Düsseldorf, Düsseldorf, Germany; 2 Senior Professorship on Work Stress Research, Medical Faculty, University of Düsseldorf, Düsseldorf, Germany; Indiana University, UNITED STATES

## Abstract

**Objectives:**

Several studies report socioeconomic differences in work stress, where people in lower socioeconomic positions (SEP) are more likely to experience this burden. In the current study, we analyse associations between education and work stress in a large sample of workers from 16 European countries. In addition we explore whether distinct national labour market policies are related to smaller inequalities in work stress according to educational attainment.

**Methods:**

We use data collected in 2010/11 in two comparative studies (‘Survey of Health, Ageing and Retirement in Europe’ and the ‘English Longitudinal Study of Ageing’; N = 13695), with samples of men and women aged 50 to 64 from 16 European countries. We measure highest educational degree according to the international standard classification of education (ISCED) and assess work stress in terms of the demand-control and the effort-reward imbalance model. National labour market policies are measured on the basis of policy indicators which are divided into (1) ‘protective’ policies offering financial compensation to those excluded from the labour market (e.g. replacement rate), and (2) ‘integrative’ policies supporting disadvantaged individuals on the labour market (e.g. investments into active labour market policies or possibilities for further qualification in later life). In addition to country-specific analyses, we estimate multilevel models and test for interactions between the indicators of national policies and individual education.

**Results:**

Main findings demonstrate consistent associations between lower education and higher levels of work stress in all countries. The strength of this association, however, varies across countries and is comparatively small in countries offering pronounced ‘integrative’ policies, in terms of high investments into measures of an active labor market policy and high participation rates in lifelong learning activities.

**Conclusions:**

Our results point to different types of policies that may help to reduce educational differences in work stress, in particular policies supporting those who are disadvantaged on the labour market.

## Introduction

Occupational health research has established solid evidence on the impact of work stress on health, mainly based on the application of theoretical models in epidemiological cohort studies. Work related stress has been found, for instance, to be associated with increased risks of cardiovascular disease [1–3], affective disorders [4–6], and a range of other health risks [7–9]. Importantly in many, but not all [10,11] studies, measures of work stress followed a social gradient, with higher levels of work stress among workers in more disadvantaged socioeconomic positions (SEP) [12–14]. This finding is relevant in the context of explaining social inequalities in health among working populations, because work-related stress may be an important intermediate factor linking low SEP with poor health. Most existing studies supporting this ‘mediation’ hypothesis, show that associations between SEP and health are generally weaker once work stress is considered in multivariate analyses [15–17].

Against this background it is important to explore socioeconomic differences in work stress in more detail, specifically in a cross-country perspective. This perspective offers the opportunity to compare associations between SEP and work stress in a variety of countries. In addition it is possible to study whether there is a link between the extent to which specific national policy regulations are implemented and the magnitude of socioeconomic inequalities in stressful work. This latter question is of particular interest in view of the explicit goal of national welfare policies to improve living and working conditions of their socially disadvantaged population groups. In this paper we set out to study this research question in the frame of a cross-national survey of older employees in 16 European countries. More specifically, we study differences in work stress between different educational groups. Work stress was operationalised in terms of two theoretical models, the demand-control model [18], and the effort-reward imbalance model [19]. While work stress in the first model is a result of a distinct job task profile, i.e. high demand in combination with low control (‘job strain’), its definition in the second model is based on the work contract: It results from high efforts spent at work not being reciprocated by appropriate rewards in terms of salary, job promotion or security, and esteem. Before describing the study design and the methods we explain the role of labour market policies and education in this context.

There is increasing evidence that the overall level of work stress in a country is related to its wider political context [20]. Existing welfare state typologies constitute an important conceptual background at this point, but recent empirical research has followed a more comprehensive approach that uses quantifiable indicators of labour market policies. For example, active labour market policies (ALMP, measured as % of GDP) were related to favourable working conditions, in particular policies that promote further qualification among adult workers and that invest in supported employment and rehabilitation services [21,22]. Furthermore, recent findings suggest that the strength of associations between work stress and employees’ mental health is generally smaller in countries with extended unemployment protections, in particular, the unemployment benefit levels [23]. These findings suggest that two types of labour market policies are of special interest, protective and integrative policies. Integrative policies aim to promote return to work in case of unemployment or support job maintenance for those in precarious employment (e.g. through ALMP or workplace training). Protective policies refer to social provision through compensation of income loss following unemployment or precarious employment (e.g. level of unemployment benefit). In terms of existing country-variations, European countries have generally experienced a substantial decrease of protective policies and an expansion of integration policies. Although the decline of protective policies has been very pronounced in case of Scandinavian countries (in particular Sweden) and continental European countries, these countries still have relatively extensive protective policies in particular compared to Anglo-Saxon countries. Similarly, albeit the expansion of integrative measures was observed in most European countries, Scandinavian countries (in particular Denmark) still have highest spending levels of ALMP followed by continental countries, and lowest levels in Anglo-Saxon and Eastern European countries [24].

Yet, an open question is whether these two types of labour market policies also have an impact on the magnitude of the socioeconomic differences of stressful work at a national level. For example, protective policies offering financial compensation in case of job loss (e.g. replacement rate) may reduce socioeconomic differences in work stress, because they offer financial security employees can rely on [25]. In contrast, as long as individuals' standard of living entirely depends on market performance, it is likely that employees are forced to accept any kind of job, even if this means that they have to work under stressful conditions. In a similar way, integrative policies supporting those who experience difficulties in entering or staying in the labour market may be important as well. For example, ALMP and lifelong learning opportunities may enhance the skills of employees with lower educational levels and promote their (re-) integration into the labour market [24]. In this study we test if national policies, in terms of protective and integrative policies, reduce socio-economic differences in work stress.

In the following analyses, we use the respondents' highest educational degree, as defined by the International Standard Classification of Education (ISCED, see Methods for details). This measure explicitly considers national variations in educational systems, therefore allowing for cross-country comparisons. Educational qualification is an important determinant of adult life chances as it provides resources and capabilities required for successful labour market integration. This includes individuals' occupational position in working life and labour market disadvantage [26]. In addition, people with lower levels of education may profit specifically from the labour market policies described above, and thus, this indicator appears appropriate for our analyses. For example, by improving the level of qualification through further education, people with lower educational classification may profit in particular. Education has the advantage of being relatively stable throughout the life course [27], and of being closely associated with a range of health conditions [28,29]. Moreover, educational qualification may moderate the effects of distinct labour market policies on work and employment [26].

## Methods

### Data source

We use cross-sectional data from two studies on ageing, with information collected in 2010/2011 in 16 European countries, the ‘Survey of Health, Ageing and Retirement in Europe’ (SHARE, 15 countries) and the ‘English Longitudinal Study of Ageing’ (ELSA). These two studies were developed in close coordination, with a focus on harmonization of research methods and study designs to allow for cross-national comparisons. In each country nationally representative samples of individuals aged 50 and older were drawn. Participants answered questions on sociological, economic and health-related topics in a face-to-face interview. In each single country, samples were drawn independently and are based on probability household samples (either drawn as simple random selection or multistage random selection) [30,31]. SHARE was launched in 2004 while the first wave of ELSA started in 2002, with on-going waves of data collection in two-year intervals in both studies (and new countries joining SHARE in later waves). New participants were added in later waves to maintain population representation. With regard to survey participation, response rates of SHARE and ELSA are generally above average compared to other European Surveys [30]. At study onset rates were 70% in case of ELSA and 61% for the total sample in SHARE ranging from 39% in Switzerland to 81% in France. By combining data from the latest waves of the two surveys, collected in 2010/2011, countries range from Northern Europe (Sweden and Denmark), Western Europe (Germany, the Netherlands, Belgium, France, Switzerland and Austria), Southern Europe (Italy, Spain and Portugal), Central and Eastern Europe (Czechia, Poland, Slovenia and Estonia) to England (In 2010/2011 data were also collected in Hungary. Following preliminary analyses we decided to exclude this country due to outlying and implausible values (extremely high inequalities in work stress according to education)).

Because we were interested in work stress, we restricted the sample to all employed men and women. Further, because people working beyond state pension age may both have better working conditions—in terms of lower levels of stress at work—and better health (healthy worker effect) we restricted the sample to men and women younger than 65. This resulted in a sample of 13695 respondents (51.7% women) aged 50 to 64 with full available data on all variables. For the analyses, calibrated weights are applied for descriptive purposes. These weights are calculated for each country separately and help to compensate for unit non-response. Details on each survey are provided elsewhere [30–34]. Participants gave written informed consent to participate in the study. SHARE was approved by the institutional review board at University of Mannheim, Germany. Ethical approval for ELSA was obtained from the Multi-Centre Research Ethics Committees in the United Kingdom.

### Measures

#### Work stress

Work stress was assessed by a short battery of items derived from (a) the Job Content Questionnaire measuring the demand-control model [35] and (b) from the effort-reward imbalance model questionnaire [36]. Given the constraints of a multi-disciplinary approach the inclusion of the full questionnaires was not possible in both surveys. Therefore abbreviated measures were used. The items were selected on the basis of factor loadings on respective original scales. In case of the demand-control model, the measurement was restricted to the control dimension based on evidence that the predictive power of control exceeded the power of demand in some studies [37]. Control was measured by the sum score of two Likert-scale items. The response categories of these items range from 1 to 4, with higher scores indicating lower control at work. The sum score of these two items varies between 2 and 8. To measure effort-reward imbalance, 2 items measuring ‘effort’ and 5 items assessing ‘reward’ at work were used. 'Effort-reward imbalance' was then calculated by dividing the sum score of the 'effort' items (nominator) through the sum score of the 'reward' items (adjusted for number of items; denominator). This ratio of the effort and reward items results in a sum score ranging from 0.25 to 4 where higher values are related to higher levels of work stress.

#### Education

Our measure of education is based on respondents' highest educational degree that we regrouped according to the International Standard Classification of Education (ISCED). This classification was developed to improve the comparability of educational attainment between different countries. Following previous procedures [28], levels of classifications were regrouped into three categories: low (level 0–2; pre-primary, primary, or lower secondary education), medium (level 3; secondary or post-secondary education), and high educational attainment (level 4–6; first and second stage of tertiary education). This is justified by the fact that the selected categories represent significant levels of educational attainments with important consequences for occupational positions in working life [38].

#### Additional measures

We included age, sex, employment status (self-employed vs. employed) and work time (full-time (>35 hours per week) versus part-time) as additional variables in the analyses.

#### Policy indicators

Rather than using existing welfare state typologies for our analyses, we use four policy indicators of national labour market policies, each provided from official sources on a coherent and comparable basis. As described in the introduction, these indicators cover two relevant dimensions of labour market policies (two for each dimensions), that is, ‘integrative’ and ‘protective’ labour market policies.

In case of ‘protective’ policies, the first measure is the so-called ‘replacement rate’. It describes the expected net income in the period directly after job loss as a percentage of the net income before job loss. Second, we use one indicator provided by the OECD that summarizes the amount of a country’s labour market expenditures into ‘passive labour market policies’ (PLMP), expressed as percentage of GDP [39]. More specifically, PLMP is divided into two sorts of expenditures, first, in investments that aim to compensate individuals temporarily for loss of wage or salary (e.g. unemployment benefit), and second, expenditures that compensate and promote premature retirement of older workers with disadvantages on the labour market.

Turning to integrative policies, we use one indicator measuring the extent of lifelong learning possibilities in a country, and a second indicator measuring the amount of investments into ALMP. In case of lifelong learning, the indicator refers to older men and women (55 to 64) who stated that they received education or training in the last 12 months (in per cent). This information is provided by EUROSTAT and was collected in the ‘Adult Education Survey’ [40]. In case of ALMP, information is again expressed as percentage of GDP and comprises various policy measures of ALMP (usually classified into 6 different types of actions), in particular interventions that aim to promote labour market integration for disadvantaged groups. These four policy indicators are described in detail in Table 1, and we present country values in the supporting information (S1 Table).

**Table 1:** Policy indicators.

**Protective labour market policies**	
PLMP ^a^	Passive labour market policies refer to public expenditures that aim to compensate individuals, both in case of (1) loss of wage or salary and (2) of involuntary early retirement. In the analysis, the indicator is measured in percentage of GDP.
Replacement rate	This measure describes the expected net income in the period directly after job loss. Measured in percentage of the net income before job loss.
**Integrative labour market policies**	
Lifelong learning	The variable refers to persons aged 55 to 64 who stated that they received education or training in the 12 months preceding the survey.
ALMP ^a^	Active labour market policies refer to public expenditures that aim to promote labour market integration for groups that are disadvantaged in the labour market (including unemployed people seeking for a new job). It comprises (1) training programs (workplace training or further education) (2) job rotation and sharing (skill enlargement) (3) employment incentives (incentives to hire new workers or maintain jobs) (4) supported employment and rehabilitation (services for people with limited working capacity) (5) direct job creation (provision of jobs in the public sector) and (6) start-up incentives (grants provided to start-up business). In the analysis, the indicator is measured in percentage of GDP.

Note. ^a^ For the analyses, we followed previous procedures [49] and weighted measures (% of GDP) according to existing unemployment rates. This prevents the possibility that country's higher expenditures were simply related to higher levels of unemployment [50].

### Statistical analyses

**Fig 1:**
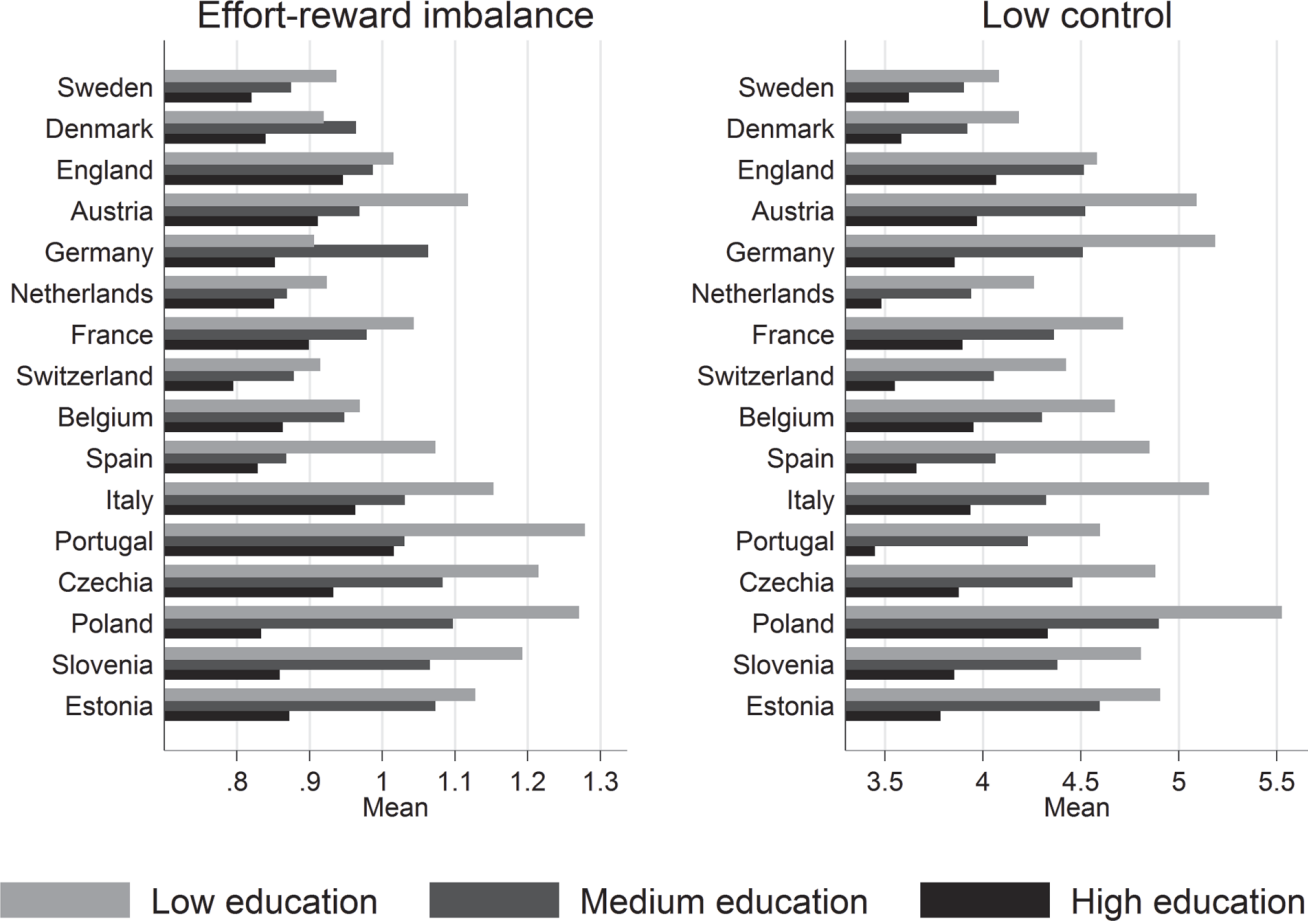
Average levels of work stress (mean score) by education and country. Note. Results are based on weighted data.

**Fig 2:**
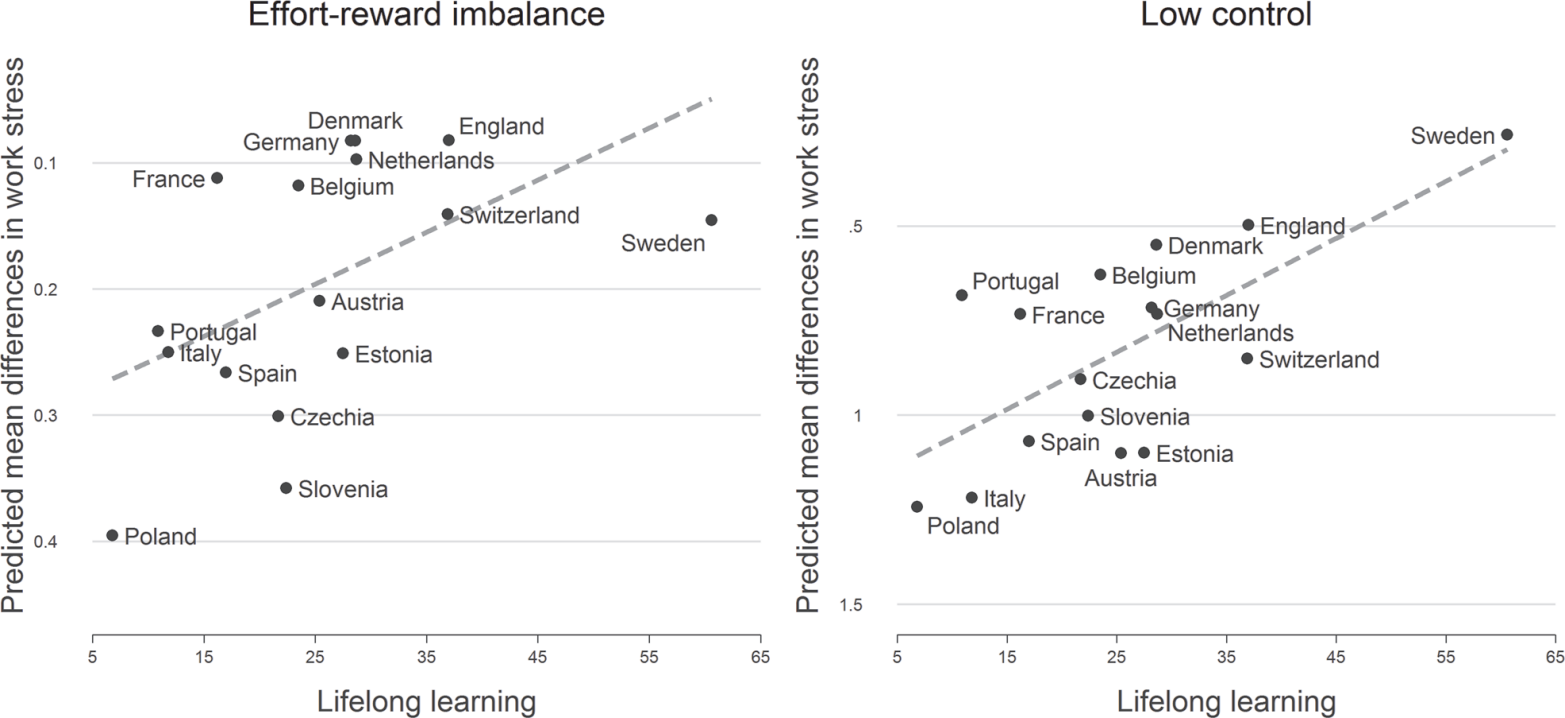
Educational differences in work stress (low vs. high education) and lifelong learning (% of older worker in further education). Note. Mean differences are adjusted for age, sex, self-employment and work time (based on Table 2).

**Fig 3:**
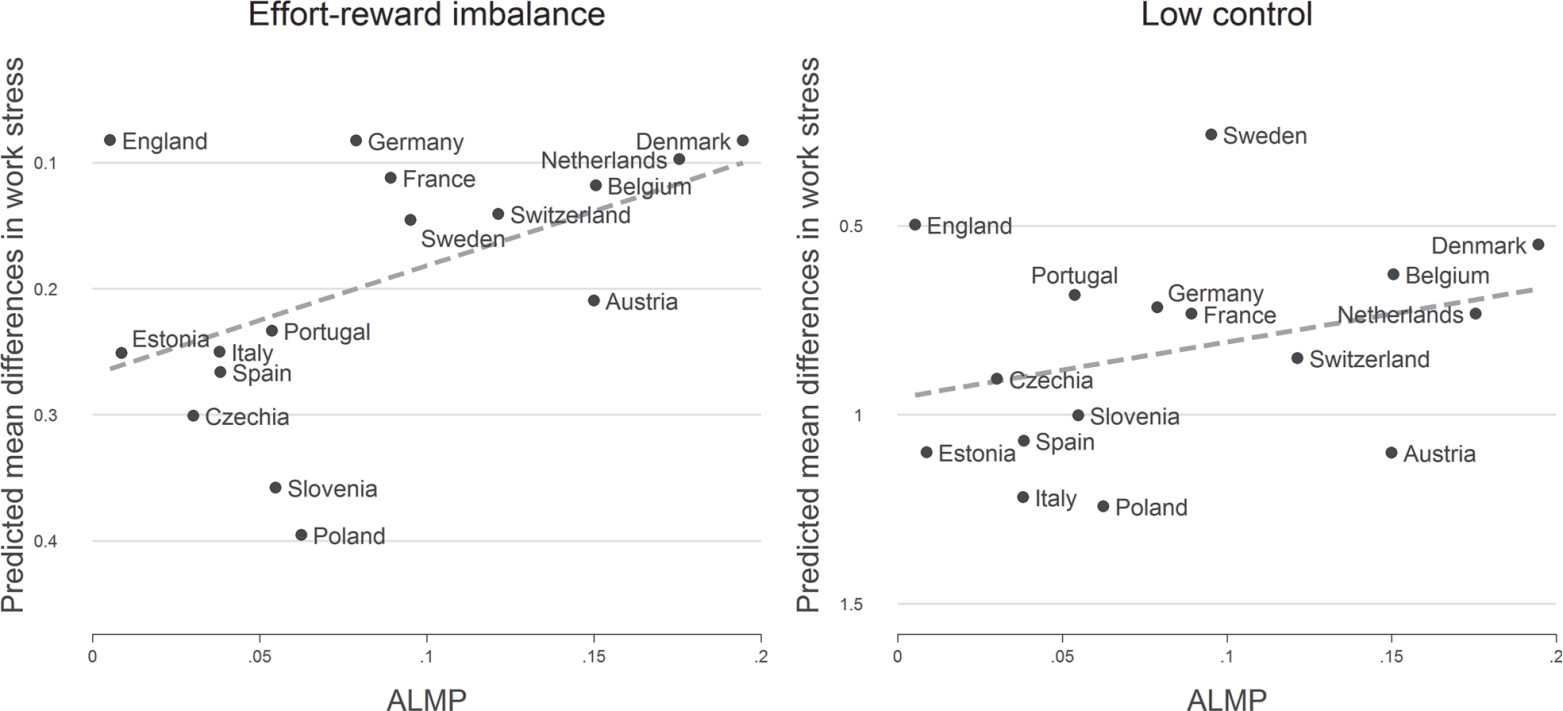
Educational differences in work stress (low vs. high education) and ALMP (expenditure into active labour market programmes). Note. Mean differences are adjusted for age, sex, self-employment and work time (based on Table 2). Expenditures into active labour market policies (ALMP) are based on % of GDP (weighted by unemployment rate).

Following a basic description of the sample (Table 2), we present two sets of analyses. The first set compares levels of work stress by educational qualification for each country. To do so, Fig. 1 presents average levels of work stress (for both work stress models) for each level of education, and we estimate multivariate linear regression models using work stress as dependent variable. More specifically, we test associations between education (broken down into dummy variables) and the two work stress measures (adjusted for sex, age-categories, employment status and work time) for each country separately. These findings are presented in Table 3, where we list the estimated coefficients for education using high education as reference category. In addition, to explore how educational differences are related to labour market policies, we study if effect sizes (as calculated within the regression models) are linked to the indicators of labour market policies, and we summarize findings in Figs. 2 and 3. Furthermore, using a subsample of the dataset with available information on occupational position in SHARE, we conduct sensitivity analyses and explore the role of occupational position within the association between education and work stress (results not presented in details). Thereby, we calculate stratified analyses (i.e. the association between education and work stress for each group of occupational position), and also estimate the association between education and work stress adjusting for occupational position. As an indicator of occupational position, we use the occupational skill level, which represents the broad hierarchical structure of the International Standard Classification of Occupation.

**Table 2:** Sample description (N = 13695).

**Variable**	**Categories or range**	**(% or mean)**	**N**
Sex	Male	48.3	6620
	Female	51.7	7075
Age group	50–54 years	34.3	4702
	55–59 years	43.4	5944
	60–64 years	22.3	3049
Effort-reward imbalance	0.25–4	0.97	13695
Low work control	2–8	4.24	13695
Education	Low	21.9	3005
	Medium	45.0	6165
	High	33.0	4525
Employment status	Self-employed	14.8	2021
	Employed	85.2	11674
Work time	Part-time	26.3	3598
	Full-time	73.7	10097
Country	England	11.1	1516
	Austria	7.1	975
	Germany	2.2	295
	Sweden	3.1	422
	Netherlands	5.1	696
	Spain	4.3	582
	Italy	4.5	619
	France	9.6	1314
	Denmark	6.2	852
	Switzerland	9.5	1300
	Belgium	9.2	1263
	Czechia	9.6	1316
	Poland	1.3	171
	Portugal	1.8	243
	Slovenia	3.4	466
	Estonia	12.2	1665

**Table 3:** Associations between education and work stress scores by country: Results of linear regression models (unstandardized regression coefficients and p-values).

	**Effort-reward imbalance score**					**Low control score**				
	Low Education		Medium Education		High Education (ref.)	Low Education		Medium Education		High Education (ref.)
	coef.	p-values	coef.	p-values		coef.	p-values	coef.	p-values	
Sweden	0.146	(0.001)	0.073	(0.059)	-	0.259	(0.123)	0.285	(0.059)	-
Denmark	0.082	(0.094)	0.136	(0.000)	-	0.550	(0.001)	0.343	(0.000)	-
England	0.082	(0.005)	0.051	(0.032)	-	0.498	(0.000)	0.419	(0.000)	-
Austria	0.210	(0.000)	0.057	(0.037)	-	1.101	(0.000)	0.493	(0.000)	-
Germany	0.083	(0.526)	0.222	(0.000)	-	0.716	(0.067)	0.576	(0.001)	-
Netherlands	0.097	(0.003)	0.018	(0.577)	-	0.733	(0.000)	0.402	(0.000)	-
France	0.112	(0.003)	0.051	(0.115)	-	0.733	(0.000)	0.404	(0.000)	-
Switzerland	0.141	(0.000)	0.084	(0.000)	-	0.851	(0.000)	0.546	(0.000)	-
Belgium	0.118	(0.000)	0.044	(0.105)	-	0.629	(0.000)	0.351	(0.000)	-
Portugal	0.234	(0.000)	0.052	(0.558)	-	0.684	(0.000)	0.168	(0.559)	-
Spain	0.266	(0.000)	0.104	(0.031)	-	1.069	(0.000)	0.367	(0.028)	-
Italy	0.250	(0.000)	0.087	(0.109)	-	1.218	(0.000)	0.591	(0.000)	-
Czechia	0.301	(0.000)	0.159	(0.000)	-	0.906	(0.000)	0.442	(0.000)	-
Poland	0.396	(0.002)	0.225	(0.038)	-	1.243	(0.001)	0.446	(0.133)	-
Slovenia	0.358	(0.000)	0.219	(0.000)	-	1.002	(0.000)	0.670	(0.000)	-
Estonia	0.251	(0.000)	0.197	(0.000)	-	1.099	(0.000)	0.799	(0.000)	-
Total	0.197	(0.000)	0.103	(0.000)	-	0.869	(0.000)	0.510	(0.000)	-

Note. All models are adjusted for sex, age groups, employment status and work time.

In a second set of analyses, we combine all countries (pooled dataset), and we study the effects of the four policy indicators in more detail, using linear multilevel regression models (random intercept only). Given the multilevel structure of the data, we estimate a multilevel model with individuals (level 1) nested within countries (level 2). Using multilevel modelling allows for accurate adjustment for country affiliation, because the intercept is allowed to vary across countries. This is important for our analyses, because of previously reported country-variations of work stress [21,41]. In addition, we conducted likelihood ratio tests to compare the multilevel models to conventional linear regression models (with country dummies), and these tests revealed better model fits in all cases. In sum we estimate two models for each policy indicator (PI). In model 1 the association of the policy indicator with work stress is estimated, adjusting for all individual characteristics (sex, age-categories, employment status, work time and education). This procedure accounts for potential effects due to differential population composition across countries.

In model 2 we additionally include two cross-level interactions, one for medium education (denoted medium education * PI) and another one for low education (low education * PI).

**Fig 4:**
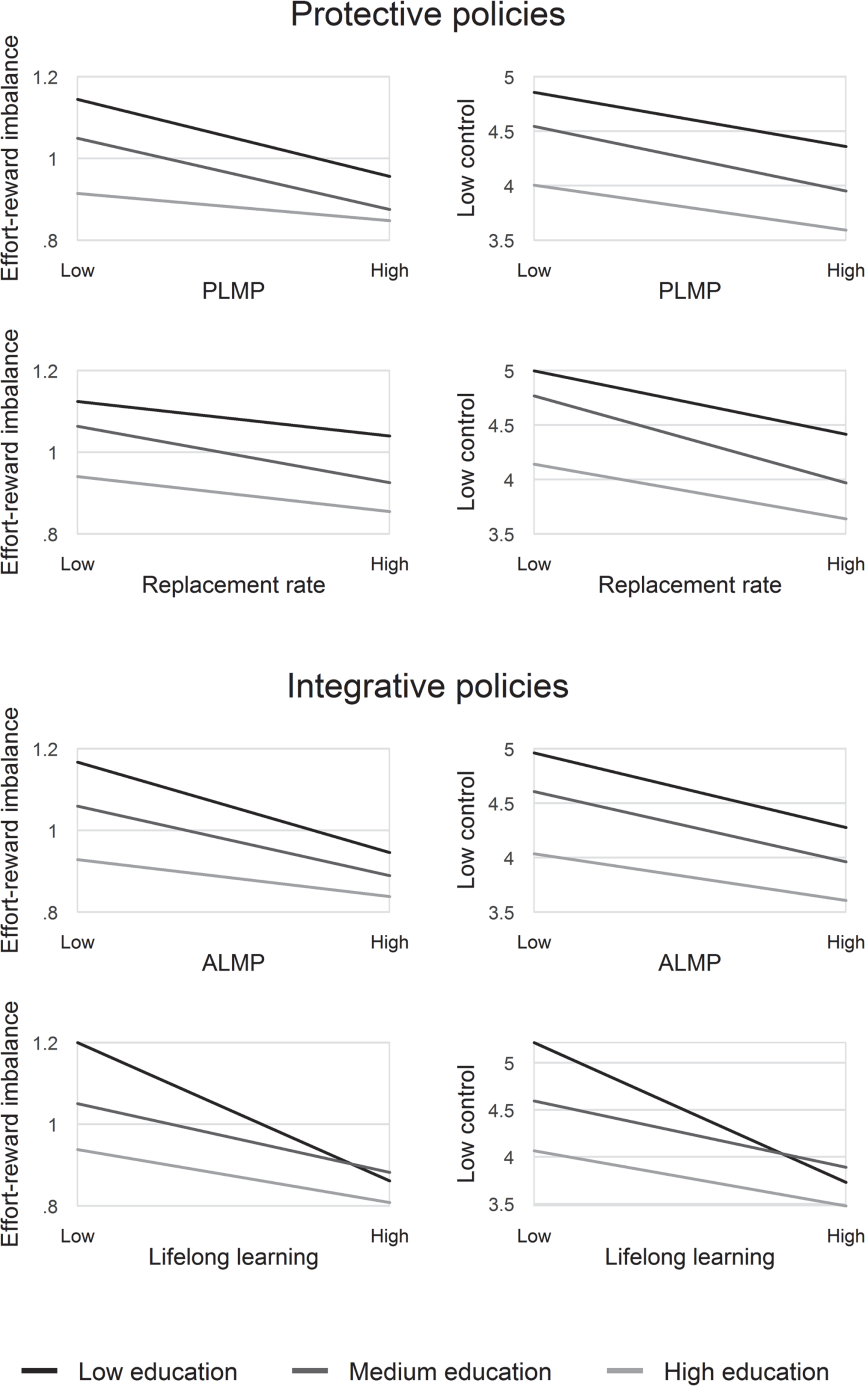
Predicted levels of work stress by education at different levels of policy indicators. Note. Expenditures into active (ALMP) and passive labour market policies (PLMP) are weighted by unemployment rate. Results are based on Table 4, model 2.

With these two interaction terms we seek to answer our core research question, i.e. whether there are significant differences in the effect size of the policy indicator on work stress between the medium education group and the low education group respectively as compared to the high education group (reference category). As a formal test of significance, we also apply a likelihood ratio test (LR Test) that indicates whether the two interaction terms add explanatory power to the one displayed in model 1. The results of these analyses are displayed in Table 4, where we present the effects for each policy indicator and additionally the interactions with education. Given a rather complicated interpretation of the findings, we visualise them in Fig. 4. In this Figure, levels of work stress are predicted by educational category at different levels of policy indicators.

**Table 4:** Association between policy indicators and work stress (model 1) and interactions between education and policy indicators (model 2): Results of random intercept linear multilevel regressions. Unstandardized regression coefficients (p-values).

	Integrative policy indicators		Protective policy indicators	
	ALMP	Lifelong learning	PLMP	Replacement rate
**Effort-reward imbalance**				
**Model 1**				
Policy indicator	-0.748 (0.001)	-0.003 (0.006)	-0.376 (0.007)	-0.003 (0.196)
**Model 2**				
Policy indicator (PI)	-0.452 (0.047)	-0.002 (0.108)	-0.180 (0.217)	-0.002 (0.332)
Medium edu.* PI	-0.397 (0.002)	-0.001 (0.477)	-0.329 (0.000)	-0.001 (0.163)
Low edu. * PI	-0.654 (0.000)	-0.003 (0.001)	-0.291 (0.000)	-0.000 (0.987)
LR test	<0.001	0.002	<0.001	0.311
**Low control**				
**Model 1**				
Policy indicator	-2.846 (0.001)	-0.014 (0.001)	-1.375 (0.012)	-0.016 (0.030)
**Model 2**				
Policy indicator (PI)	-2.151 (0.015)	-0.009 (0.037)	-1.118 (0.049)	-0.013 (0.108)
Medium edu. * PI	-1.068 (0.008)	-0.002 (0.496)	-0.485 (0.050)	-0.007 (0.012)
Low edu. * PI	-1.280 (0.013)	-0.014 (0.000)	-0.227 (0.446)	-0.002 (0.636)
LR test	0.009	<0.001	0.146	0.036

Note. All models are adjusted for sex, age groups, employment status and work time. Expenditures into active (ALMP) and passive labour market policies (PLMP) are weighted by unemployment rate.

## Results

### Sample description

There are slightly more women participating (N = 7075) than men (N = 6620), with a mean age of 56.4 years. A minority of participants only is self-employed (15%) or working in part-time jobs (26%). The average number of observations across countries is 856, with the smallest number in Poland (171) and largest number in Estonia (1665). Most respondents (45%) have secondary or post-secondary education as highest educational degree (medium education), while 33 per cent entered tertiary education (highest degree) (see Table 2 for details).

### Educational differences in work stress by country

Fig. 1 displays associations between education and both measures of work stress. First, in each country we observe that people with a lower educational level experience a higher amount of stressful work, thus supporting the notion of a social gradient of work stress. Second, educational differences appear to be more pronounced in Eastern European countries (especially Slovenia and Poland), compared to those observed in Northern Europe, where England, Sweden and Denmark seem to have smaller educational differences. These latter findings are confirmed by the results of multivariate models presented in Table 3. In all countries, we observe positive coefficients for respondents with low education, thus indicating higher levels of work stress as compared to respondents with high education. Respective differences are significant for either work stress model in 14 out of 16 countries, even after adjusting for sex, age, employment status and work time. In additional sensitivity analyses based on a subsample, the association between education and work stress was also consistent across different groups of occupational position.

Turning to our core research question, that is, variations of effect sizes between countries, we again observe that educational differences are particularly pronounced in Eastern Europe while remaining relatively small in Scandinavian countries (with insignificant differences in case of effort-reward imbalance for Denmark and in case of low control for Sweden). How are these differences related to national policies, and in particular, to integrative and protective policies? Before presenting results of multilevel findings testing for respective interactions, we briefly describe the comparisons between estimated coefficients and country-values for the four policy indicators under study (listed in S1 Table): While there is support for the assumption that the two indicators of ‘integrative’ policies (ALMP and lifelong learning) are related to educational differences, associations between educational differences and ‘protective’ policies are less apparent. In particular, countries with pronounced measures of active labour market policy (ALMP) and with high participation rates in lifelong learning activities display smaller differences in work stress on the basis of educational attainment than countries with less developed policy measures. These latter findings are summarized in Fig. 2 for lifelong learning and in Fig. 3 for ALMP.

### Findings of multilevel models

Table 4 shows the results of the multilevel models, testing whether the strength of associations between educational level and work stress varies according to the extent of implementation of each one of the four policy indicators. We first study if the four policy indicators are related to overall levels of work stress (model 1), and second, if effect sizes of the policy indicators vary by level of education (model 2). Taken together, the two following observations deserve attention: First, we find a negative and significant association between all indicators of ‘integrative’ policies (ALMP and lifelong learning) and the two measures of work stress. For instance, countries with pronounced active labour market policies (ALMP) have lower levels of work stress. To a lesser extent, the two indicators of ‘protective’ policies (PLMP and replacement rate) were related to lower levels of work stress, with significant results in three out of four cases. Second, once we include cross-level interactions in model 2 (to test if effects of the policy indicators vary by education) we observe that these interactions are significant in most cases, in particular for the two measures of ‘integrative’ policies (Table 4). Patterns are similar for both models of work stress. This result may suggest that participants with a low educational level benefit more from such policy measures in terms of lowering their burden of work-related stress than participants with higher educational qualification.

### Predicted levels of work stress by education and policy measures

Fig. 4 visualizes the main results of model 2, where levels of work stress are predicted for each educational group according to the extent of policy development for each one of the four policy indicators. Again, we see that levels of work stress are generally higher if ‘integrative’ or ‘productive’ policies are less developed. In addition, the steepness of educational differences in stressful work varies according to the amount of policy development, such that differences tend to diminish if national policies are well developed. This is most obviously demonstrated in case of the indicator ‘lifelong learning’ reflecting an important integrative labour market policy measure.

## Discussion

In this contribution differences in work stress in relation to educational attainment among older employees (50 to 64 years) were studied in a cross-country perspective, using data from two European surveys covering 16 countries (SHARE and ELSA, N = 13695). Three findings were of particular interest. First, we confirmed a rather robust social gradient of stressful work in terms of low control and effort-reward imbalance, where those with lower educational qualification had higher levels of stressful work. Second, in countries with well-developed labour market policies average levels of work stress were generally lower than in countries with less developed policies. Third, the steepness of educational differences in stressful work varied according to the extent of policy development such that differences tended to be smaller if national policies were well developed. This pattern was similar for both models of work stress. The associations between policy indicators and educational differences in stressful work were more consistent in case of ‘integrative’ vs. ‘protective’ policies.

While the first result corroborates previous findings it adds a new element as we use education instead of occupational position to investigate the social gradient of work stress [12,13,27]. As shown in previous studies, education is a core marker of SEP and has high significance for unequal life chances including occupational careers [26]. Along these lines, additional sensitivity analyses also explored the role of occupation in more details (as described in the methods section). In these analyses we found support of an association between education and work stress within each occupational position. Also, we found that effects of education on work stress are generally reduced, but remain significant after adjustment for occupational position in multivariate models. These findings lend important support in favor for an indirect effect of education through occupational position, but it also suggests that the effect of education is only partly due to occupational position.

Our second finding is in accordance with previously documented associations of average quality of work of a country’s working population with the extent to which distinct indicators of national social and labour market policies are implemented [21,22]. However, our analyses are based on two conceptually distinct sets of policy measures, labeled ‘integrative’ and ‘protective’ policies. This approach is in line with recent developments within welfare state literature [41,42,43]. It offers the opportunity of exploring whether the former, more active, integrative policies are more responsive to the needs of socially disadvantaged groups than is the case for ‘protective’ policies [42]. This latter assumption has been tested with regard to educational differences in the amount of stressful work where our third finding demonstrates smaller social gradients of stressful work under conditions of well developed ‘integrative’ labour market policies. One possible explanation of this finding is that ‘integrative’ labour market policies enable individuals to find better jobs and therefore improve working conditions [44]. This was especially evident in case of the indicator ‘lifelong learning’. To our knowledge, this hypothesis has not been tested so far. Our findings suggest that efforts to further qualify workers even in later phases of their career and help to successfully integrate them into the labour market may buffer the negative consequences of a low formal education gained in adolescence or early adulthood. Yet, while less important in reducing educational differences, our findings nevertheless show that ‘protective’ policies matter as well, in particular in reducing the overall level of work related stress. Similarly a recent study also showed that ‘protective’ policies may mitigate the effect of work stress on health [23].

This study has several limitations. First, our measurement of work stress was restricted to short assessments of single components of two established theoretical models, demand- control and effort-reward imbalance. ELSA and SHARE, the two studies used in our analyses, only include the control scale of the demand control model. It is therefore not possible to define job strain, the combination of high demands and low control, in our analyses. A stronger case would have been provided if the full original questionnaires were applied and if additional aspects of stressful work were incorporated [45,46]. Second, our choice of indicators of national labour and social policies can be challenged both in terms of coverage of relevant policy domains and in terms of accuracy of reflecting the specific quality of national policy developments. Third, although our study includes a variety of countries with different historical and political background, a larger number of countries would have strengthened the case and would correspond more appropriately to the methodological requirements of applying multilevel statistical analysis. Fourth, the cross-sectional design of the study prevents any conclusion concerning the direction of observed statistical effects. For instance, we do not know whether the implementation of a productive national policy measure is followed by a subsequent reduction in work stress or in an attenuation of its social gradient. The study population consists of of men and women aged 50 to 64. This age group is very sensitive to the healthy worker effect which could have affected the results [47], and therefore, future studies may replicate our findings to other age groups as well. Finally, while the overall sample size of this study is quite large, it is rather low in some of the countries included. On the one side, it is probable that this may affect the composition of our sample and thus the overall level of work stress therefore calling for further validation. Yet, on the other side, it seems less probable that low response rates additionally affect the associations under study and its strength.

Despite these limitations the study has several strengths. It is based on two prominent, internationally established models of stressful work. It identifies national social and labour policies at the level of quantified, cross-nationally comparable indicators. Moreover, our choice of indicators reflects a recent conceptual shift in welfare state research from ‘protective’ to ‘integrative’ policies activating and integrating disadvantaged groups of the labour market [42]. In this context we tested an innovative research hypothesis and found preliminary empirical support. Finally, the availability of a large comparative data set from two European studies enabled us to study variations of national policies and their relevance for average work stress levels in these countries and their social gradient. Previous research found preliminary evidence that distinct labour policies can mitigate the effects of stressful work stress on mental health at the level of respective populations [23].

If the findings of this study represent robust trends in further analyses, they may have important policy implications. More specifically, our main result suggests that distinct ‘integrative’ labour market policies exert more beneficial effects (in terms of lowering average levels of work stress) among socially disadvantaged groups than is the case for passive or ‘protective’ policies. This is eventually due to the fact that these policies target the needs of populations suffering from labour market disadvantage more effectively than other policies [20]. Therefore they may offer a promising entry point for policies that aim at reducing social inequalities in work and employment and their adverse effects on health and well-being [48].

In conclusion, this study illustrates that people with lower educational qualification are more likely to experience work-related stress in a range of European countries, and that specific national labour policies may contribute to a reduction of the social gradient of stressful work in respective countries. Given the negative consequences of work-related stress on mental and physical health, our findings can direct political attempts towards reducing social inequalities in the health of working populations.

## Supporting Information

S1 Table Macro indicators by country. Note. ALMP and PLMP measures in parentheses are weighted (ALMP or PLMP / unemployment rate)
(DOCX)
