# Supplementary material for: The Association between Education and Work Stress: Does the Policy Context Matter?
Source: PLoS One. 2015 Mar 26;10(3):e0121573. doi: 10.1371/journal.pone.0121573 (PMC4374794; doi:10.1371/journal.pone.0121573)
Supplement: S1 Table — Note. ALMP and PLMP measures in parentheses are weighted (ALMP or PLMP / unemployment rate) (DOCX) [file pone.0121573.s001.docx]

Table S1 Macro indicators by country

|  | Integrative | |  | Protective | |
| --- | --- | --- | --- | --- | --- |
| Country | ALMP (weighted) | Lifelong Learning |  | PLMP (weighted) | Replacement rate |
| Sweden | 0.8 (0.095) | 60.6 |  | 0.8 (0.095) | 67.25 |
| Denmark | 1.44 (0.195) | 28.6 |  | 1.78 (0.241) | 83.63 |
|  |  |  |  |  |  |
| England | 0.04 (0.005) | 37.0 |  | 0.30 (0.039) | 60.88 |
|  |  |  |  |  |  |
| Austria | 0.66 (0.150) | 25.4 |  | 1.40 (0.318) | 66.13 |
| Germany | 0.56 (0.079) | 28.2 |  | 1.33 (0.187) | 66.88 |
| Netherlands | 0.79 (0.176) | 28.7 |  | 1.75 (0.389) | 79.63 |
| France | 0.83 (0.089) | 16.2 |  | 1.45 (0.156) | 70.00 |
| Switzerland | 0.51 (0.121) | 36.9 |  | 0.78 (0.186) | 83.00 |
| Belgium | 1.25 (0.151) | 23.5 |  | 2.26 (0.272) | 72.75 |
|  |  |  |  |  |  |
| Portugal | 0.58 (0.054) | 10.9 |  | 1.39 (0.129) | 76.13 |
| Spain | 0.77 (0.038) | 17.0 |  | 3.15 (0.157) | 72.00 |
| Italy | 0.32 (0.038) | 11.8 |  | 1.45 (0.173) | 69.00 |
|  |  |  |  |  |  |
| Czechia | 0.22 (0.030) | 21.7 |  | 0.37 (0.051) | 74.50 |
| Poland | 0.6 (0.063) | 6.8 |  | 0.34 (0.035) | 69.63 |
| Slovenia | 0.40 (0.055) | 22.2 |  | 0.67 (0.092) | 81.63 |
| Estonia | 0.14 (0.009) | 27.5 |  | 0.87 (0.054) | 56.88 |

Note. ALMP and PLMP measures in parentheses are weighted (ALMP or PLMP / unemployment rate)
